# Porous and Magnetic Molecularly Imprinted Polymers via Pickering High Internal Phase Emulsions Polymerization for Selective Adsorption of λ-Cyhalothrin

**DOI:** 10.3389/fchem.2017.00018

**Published:** 2017-03-28

**Authors:** Yunlong Wu, Yue Ma, Jianming Pan, Runxing Gu, Jialu Luo

**Affiliations:** School of Chemistry and Chemical Engineering, Jiangsu UniversityZhenjiang, China

**Keywords:** pickering high internal emulsions, adsorption, λ-cyhalothrin (LC), magnetic molecularly imprinted polymers (MMIPs), porous polymers

## Abstract

A novel macroporous magnetic molecularly imprinted polymer (MMIPs) of was prepared by W/O Pickering (high internal phase emulsions) HIPEs polymerization, and then it was adopted as adsorbent for selective adsorption of λ-cyhalothrin (LC). In static conditions, adsorption capacity of LC increased rapidly in the first 60 min and reached to equilibrium in ~2.0 h. Excellent conformity of the second-order model confirmed the chemical nature of the interaction between the LC and imprinted sites. The fitting adsorption isotherm was a Langmuir type, and the maximum monolayer adsorption capacity at 298 K was 404.4 μmol g^−1^. Thermodynamic parameters suggested the specific adsorption at 298 K was an exothermic, spontaneous, and entropy decreased process. Competitive recognition studies of the MMIPs were performed with diethyl phthalate (DEP) and the structurally similar compound fenvalerate (FL), and the MMIPs, which displayed high selectivity for LC.

## Introduction

In recent years, pyrethroids are high effective insecticide widely used in agriculture, such as fishery, vegetables, forestry, and grain (Ye et al., 2015). They can be inadvertently released into the environment, resulted in contaminating rivers and ground waters. Moreover, researchers found that the presence of traces of pyrethroids in the environment posed potential neurotoxicity (Ueyama et al., 2015). Therefore, it is necessary to develop an efficient and rapid method to remove the pyrethroids released into our environment even if in the form of trace amount in water samples.

Molecularly imprinted polymers (MIPs) as a versatile technology contain highly cross-linked polymer matrix with template molecules. After the template is eluted, recognition sites in synthetic polymers exhibit a high selectivity for rebinding the target molecules and the similar structures (Chen et al., 2015; Randa et al., 2015). MIPs possess great application in a various of areas, such as drugs delivery (Li et al., 2014), biosensors (Altintas et al., 2015; Wang et al., 2016), and separation science (Takuya et al., 2015), owing to the advantages of easier preparation and high selectivity and low cost. At present, there are several studies focused on the MIPs as novel adsorbent for specific removal of antimicrobial drugs, phenols, and heavy metal ions (Pan et al., 2012; Dolak et al., 2015; Zamora-Gálvez et al., 2016).

However, the conventional MIPs have many disadvantages, for example, the precipitation polymerization exhibited low binding capacity and affinity sometimes limits their application (Wang et al., 2015). Recently, the synthesis of porous MIPs via a convenient and controllable approach has been attracted more attention due to its intrinsic advantages, such as good adsorption capacity, rapid binding kinetics, easy, and complete removal of template molecules (Qin et al., 2016). Li et al. (2014) reported a hollow porous MIPs for determination of bisphenol A in tap water using surface imprinting technique (Zhang et al., 2012). Lu et al. developed MIPs membranes with ordered porous structure via the water-assisted method (Lu et al., 2007). Zhao et al. designed a porous structure in MIPs sensor for highly selective and sensitive electrochemical determination of brucine (Zhao et al., 2014).

High internal phase emulsions (HIPEs) are characterized by possessing a volume fraction of the disperse phase that exceeds 74.05% (Lissant, 1966). It has been used as an versatile and convenient method for preparing macroporous polymers, which were named as polyHIPEs (Menner et al., 2006). However, conventional polyHIPEs have poor mechanical properties and low permeability in spite of their open-cell structure (Cameron, 2005; Menner and Biamarck, 2006). Pickering HIPEs, stabilized simultaneously by particles, have been the important templates for preparing porous foams with good mechanical property and high permeability (Ikem et al., 2010). Recently, our group has successfully fabricated imprinted polymers via W/O Pickering HIPEs polymerization for recognition of λ-cyhalothrin (LC; Pan et al., 2016c). The as-prepared MIPs with well-defined open-cell structure and interconnecting pores possessed excellent adsorption capacity and selectivity, yet the collection of them suffered from tedious after-treatment process including centrifugation or filtration which was rather time-consuming and inconvenience. Generally, magnetic MIPs (MMIPs), synthesized by coating imprinted polymer layer on the surface of magnetic material, could be simply collected from the matrix by applying an external magnetic field (Han et al., 2016; Tang et al., 2016). Our group presented the preparation of MMIPs microspheres via attapulgite and few Fe_3_O_4_ particles stabilized Pickering emulsion polymerization (Pan et al., 2013). This work inspired us to fabricate porous MMIPs by Fe_3_O_4_ particles stabilized Pickering HIPEs.

In this work, the porous MMIPs with well-defined open-cell structure were obtained through W/O Pickering HIPEs template, in which the oleic acid modified Fe_3_O_4_ particles were dispersed at the water/oil interface. Particularly, the effects of internal phase fraction, the amounts of monomer, and modified Fe_3_O_4_ nanoparticles on the foam morphology were investigated. And then porous MMIPs were employed to selective adsorption of LC, and their efficient adsorption and enhanced selectivity were discussed.

## Materials and methods

### Materials

λ-Cyhalothrin (LC), fenvalerate (FL) were supplied by Jiangsu Huangma Agrochemicals Co., Ltd. Fe_3_O_4_ nanoparticles (mean diameter is 250 nm), styrene (St), divinylbenzene (DVB), acrylamide (AM), oleic acid (OA), methacrylic acid (MAA), α,α′-azoisobutyronitrile (AIBN), toluene, methanol, acetone, chloroform, diethyl phthalate (DEP), and calcium chloride dihydrate (CaCl_2_·2H_2_O) were purchased from Sinopharm Chemical Reagent Co., Ltd (Shanghai, China). Hypermer 2,296 was purchased from CRODA UK. All chemicals were of analytical reagent grade. Water was freshly deionized and distilled.

### Instruments

Fourier transmission infrared spectra (FT-IR) of MMIPs and MNIPs were recorded on a Nicolet NEXUS-470 FT-IR apparatus (USA). UV-vis adsorption spectra were obtained with a UV-vis spectrophotometer (UV-2450, Shimadzu, Japan). The morphology of materials was obtained by scanning electron microscope (SEM, JEOL, JSM-7001F). Thermogravimetric analysis (TGA) was carried out using a DSC/DTA-TG (STA 449C Jupiter, Netzsch, Germany) under a nitrogen atmosphere up to 800°C with a heating rate of 5.0°C min^−1^. The optical micrographs of Pickering HIPEs were collected by a DMM-330C optical microscope equipped with a high performance digital camera (CAIKON, China). High-performance liquid chromatography (HPLC) analysis was performed on an Agilent system (Agilent, 1200, Germany) equipped with a UV-vis detector.

### Preparation of MMIPs

Prior to emulsification, hydrophilic Fe_3_O_4_ nanoparticles were surface functionalized with oleic acid as described in previous work (Ikem et al., 2010). Briefly, 1.0 g of hydrophilic Fe_3_O_4_ nanoparticles was immersed in a mixture of chloroform and oleic acid (OA; 1: 2, V/V). After stirring for 3.0 h, the product was filtered and washed with methanol and then the solid powders (Fe_3_O_4_-OA) were dried at 120°C for 24 h.

**Figure 1:**
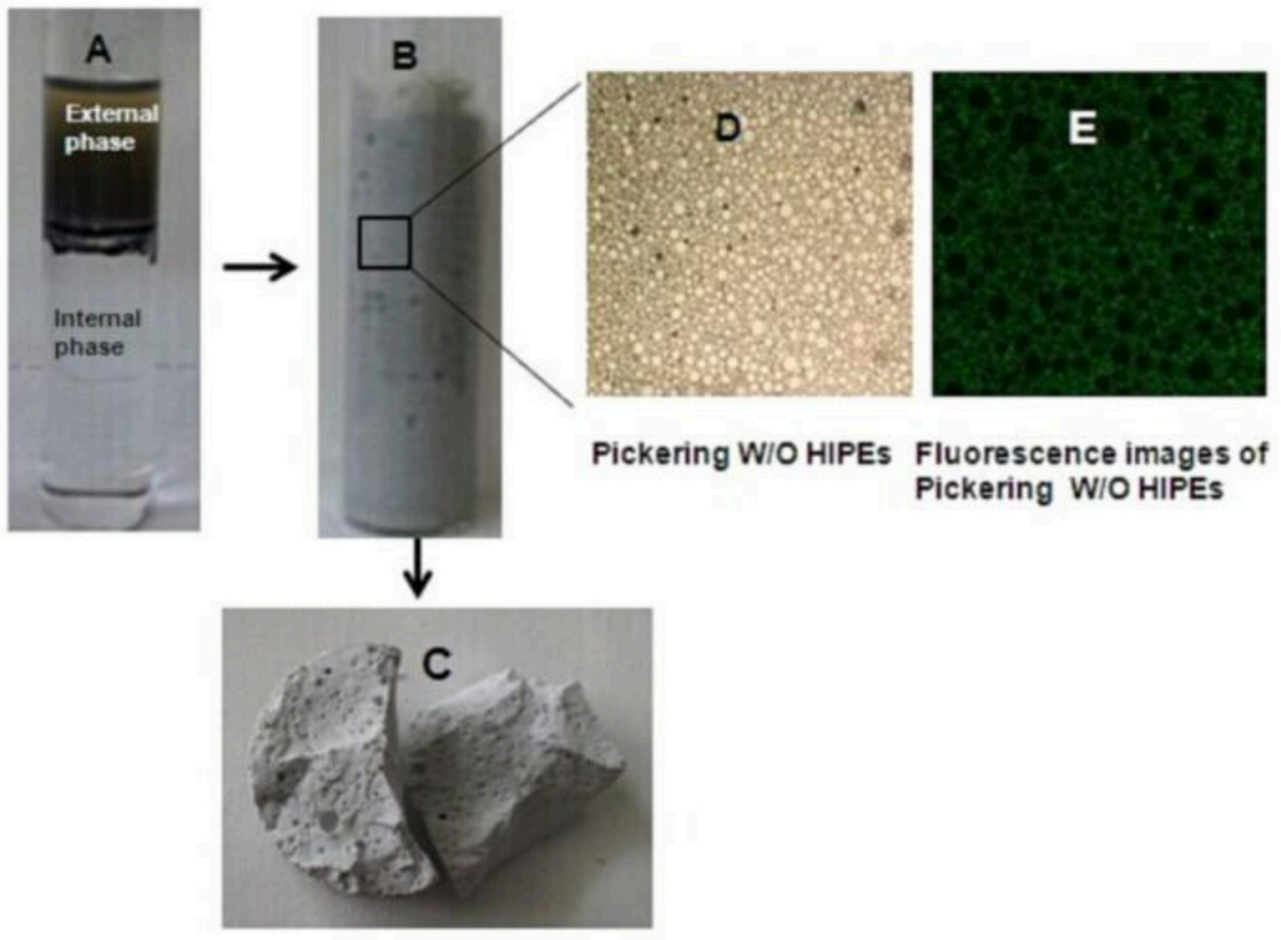
**Illustration for preparing W/O Pickering HIPEs and MMIPs. (A)** Phase-separated system, **(B)** W/O Pickering HIPEs with 85% internal phase fraction, **(C)** MMIPs monolith, **(D)** micrograph of Pickering HIPEs, and **(E)** fluorescent image of W/O Pickering HIPEs.

Illustration for the preparation of Pickering HIPEs and MMIPs was shown in Figure 1. St, DVB, AM, MAA, AIBN, Hypermer 2296, and LC were added into a three neck round bottomed flask equipped with a mechanical stirrer, and then the formed organic (continuous) phase was stirred at 400 rpm. Subsequently, aqueous (internal) phase, composed of water containing CaCl_2_·2H_2_O (0.27 M) was added drop wise to the organic phase. After addition of the internal phase, stirring for the mixture was continued for further 10 min, followed by the transfer of resultant Pickering HIPEs to an ampere bottle and cured at 70°C for 24 h. The resulting bulk polymers was purified via Soxhlet extraction with distilled water and methanol-acetic acid solution (V: V = 9:1), and then the final products (MMIPs) were subsequently dried in the oven at 120°C until the weight was constant. For comparison, the corresponding magnetic and non-imprinted polymer (MNIPs) was also prepared in the same way in the absence of LC. In order to study the effect of Fe_3_O_4_-OA, St, Hypermer 2296 and the phase volume, details of compositions prepared according to the above were summarized in Table 1.

**Table 1:** **Composition of emulsion templates**.

**Adsorbents^a^**	**Internal phase volume^b^ (%)**	**St (mL)**	**Hypermer 2296 (mL)**	**Fe_3_O_4_-OA (g)**	**Adsorption amount^c^ (μmol g^−1^)**
MMIPs 1	75	2.0	1.0	0.10	34.16
MMIPs 2	80	2.0	1.0	0.10	59.20
MMIPs 3	85	2.0	1.0	0.10	68.88
MMIPs 4	90	2.0	1.0	0.10	47.68
MMIPs 5	85	1.0	0.5	0.10	63.72
MMIPs 6	85	1.5	0.75	0.10	89.75
MMIPs 7	85	1.0	0.75	0.10	99.81
MMIPs 8	85	2.0	0.75	0.10	99.55
MMIPs 9	85	1.5	0.5	0.10	99.71
MMIPs 10	85	1.5	1.0	0.10	73.63
MMIPs 11	85	2.0	1.0	0	43.80
MMIPs 12	85	2.0	1.0	0.20	60.10
MMIPs 13	85	2.0	1.0	0.40	79.36
MMIPs 14	85	1.0	1.0	0.10	91.76
MMIPs 15	85	2.0	0.5	0.10	68.54
MNIPs	85	1.0	0.75	0.10	45.16

a *For all the samples, AM was 0.04 g, MAA was 0.04 mL, DVB was 2.0 mL, LC was 0.05 g (LC was 0 for MNIPs), AIBN was 0.06 g, and the concentration of of CaCl _2_·2H_2_O solution was 0.27 M*.

b *Internal phase consisted of the solution of CaCl_2_·2H_2_O, and the external (oil) phase consisted of St, DVB, LC, AM, AIBN, and MAA. For the calculation of internal phase volume fraction, the increase of the external (oil) phase volume due to the addition of Hypermer 2296, LC, AM, and AIBN were not considered*.

c *In a batch mode experiment, the initial concentraton of LC was 100 mg L^−1^, the weight of adsorbents was 10 mg, and the contact time was 12 h*.

### Batch mode binding experiments

In batch studies, 10 mg of MMIPs or MNIPs were added into 10 mL of LC solution with different initial concentrations (10, 30, 50, 80, and 100 mg L^−1^) in ethanol and deionized water (5:5, V/V) at 298, 318, and 338 K, respectively, and then the testing tubes were shaken at a speed of 100 rpm. After adsorption for 12 h, the adsorbents were isolated by external magnetic field. The concentration of LC in the supernatant was determined by a UV-vis spectrophotometer at 278.5 nm.

In the adsorption kinetic experiments, 10 mg of MMIPs and 10 mL of LC solutions were maintained at 298 K for different contact times (10–720 min). After the desired contacting time, the dispersions isolated by external magnetic field, and then the MMIPs were removed and the concentration of LC was detected. Moreover, the equilibrium adsorption capacity (*Q*_*e*_, mg L^−1^) was calculated by Equation (1):

(1) $$\begin{array}{r} Q_{e}=\frac{\left(C_{0}-C_{e}\right)V}{W} \end{array}$$

where *C*_0_ (mg L^−1^) and *C*_*e*_ (mg L^−1^) are initial and equilibrium LC concentrations, respectively. *V* (mL) and *W* (g) are the solution volume and the adsorbent weight, respectively.

### Binding specificity experiments

To measure the specificity of MMIPs, 10 mg of the MMIPs or MNIPs were added into 10 mL of single solution with 100 mg L^−1^ of LC, FL, and DEP, respectively. After binding experiments, the concentration of LC, FL, and DEP in the supernatant were determined by a UV vis spectrophotometer at 278.5, 277.5, and 275 nm, respectively.

### Regeneration experiments

Ten milligrams of MMIPs was firstly added into 10 mL of 200 mg L^−1^ LC solution for 12 h at 25°C, and then the amount of LC in the supernatant was determined. Secondly, MMIPs captured LC was washed by methanol/acetic acid (95:5, v/v) solution until no LC was detected in eluent. This adsorption-desorption process was repeated at least three times.

## Results and discussion

### Characterization

**Figure 2:**
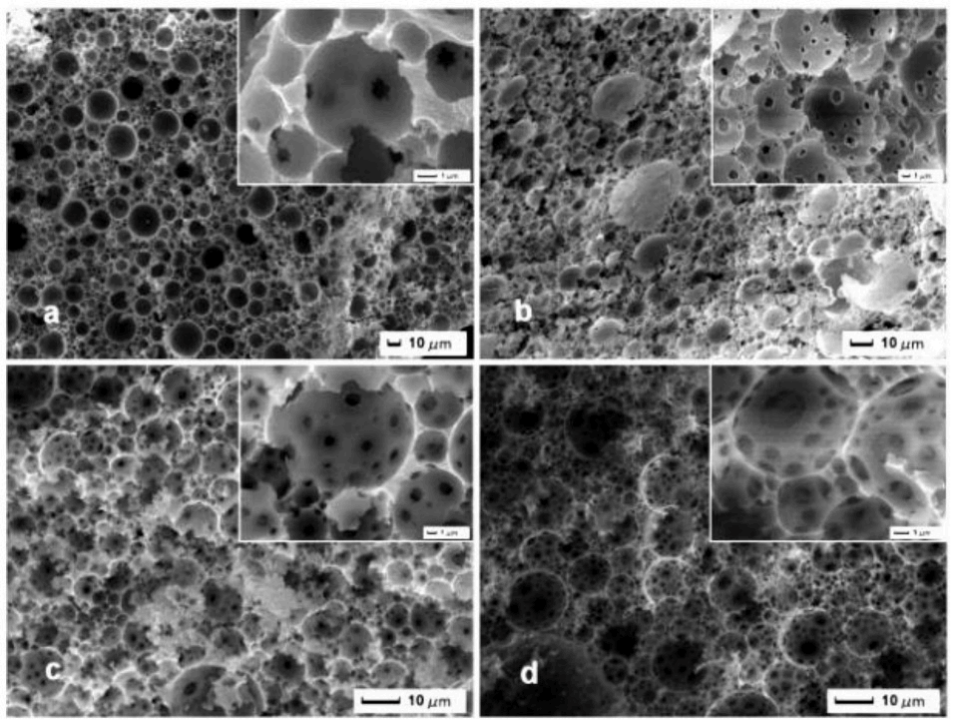
**SEM images of MMIPs 1-4 synthesized from (a)** 75%, **(b)** 80%, **(c)** 85%, and **(d)** 90% internal aqueous phase emulsion templates, and all emulsions were stabilized by 3 wt% Fe_3_O_4_-OA particles.

**Figure 3:**
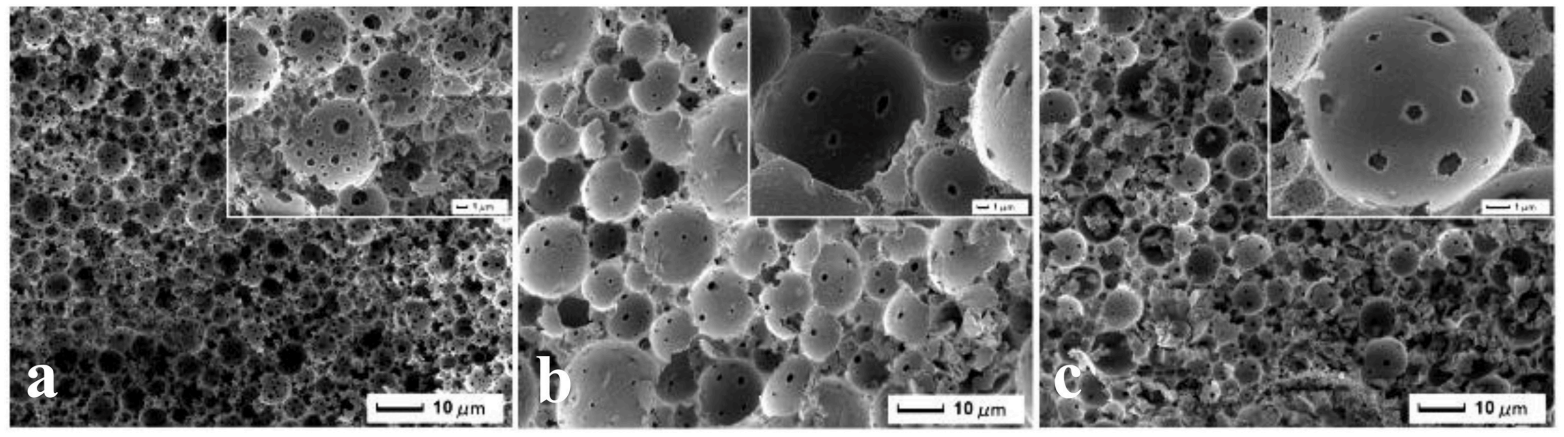
**SEM images of MMIPs 11, 12, and 13 (all samples containing surfactant and 85 vol% internal phase volumn), (a)** 0% Fe_3_O_4_ particles, **(b)** 5% Fe_3_O_4_ particles, and **(c)** 10% Fe_3_O_4_ particles, respectively.

SEM image was used to evaluate the foam morphology of the resulting brittle and light brown marcoporous polymer monolith after cutting into small pieces. It was interesting that all samples exhibited a macroporous open-cell structure with different window and pore throat in Figure 2. The polymerization of Pickering HIPEs templates with the internal phase volume of 75, 80, 85, 90%, respectively, resulted in MMIPs 1–4 (Table 1), which have many larger pores and pore throats (Figures 2a–d). These macroporous polymers have an average pore size of around 10 μm and the pore throat sizes in the range 0.5–2 μm (Figures 2a–d). It can also be seen that the size of the pore throat and windows increase with elevation of the internal phase volume, and the number of pore throat also increases gradually. As shown in Figure 2, the smaller windows of MMIPs 1 almost have a closed-cell structure with a few pore throats but thick pore walls. Moreover, the heterogeneity of pore is observed in typical structure of MMIPs 1, 2, 3, while MMIPs 4 resulted from 90% internal phase of volume possesses a higher interconnected pore structure with homogeneous windows. It should be noted that MMIPs 1 had a smooth inner surface, but the rough surface of MMIPs 2 was benefit for rebinding LC. The viscosity of these emulsions significantly enhanced along with the increase of internal phase volume (visual observed), this phenomena is mostly caused by the formation of 3D networks. Hence, the viscosity of emulsion has a remarkable effect on pore size and spatial structure of MMIPs. Consequently, the pore size and pore throat size of macroporous polymers can be tailored by varying the internal phase volume of the Pickering emulsion templates. Upon variation of the Fe_3_O_4_-OA content to MMIPs 11, 12, 13 (Figure 3), and MMIPs 3 Figure 2c), the feature size of the pore throats was decreased. It was likely that Fe_3_O_4_-OA adsorbed at the O/W interface and the aggregated nanoparticles formed clusters. As a result, a stable thicker dense particle film could not disrupt during or after polymerization, and then the continuous organic phase film between the closest neighboring droplets did not ruptured during the polymerization or purification or, more likely, during the drying of the MMIPs.

**Figure 4:**
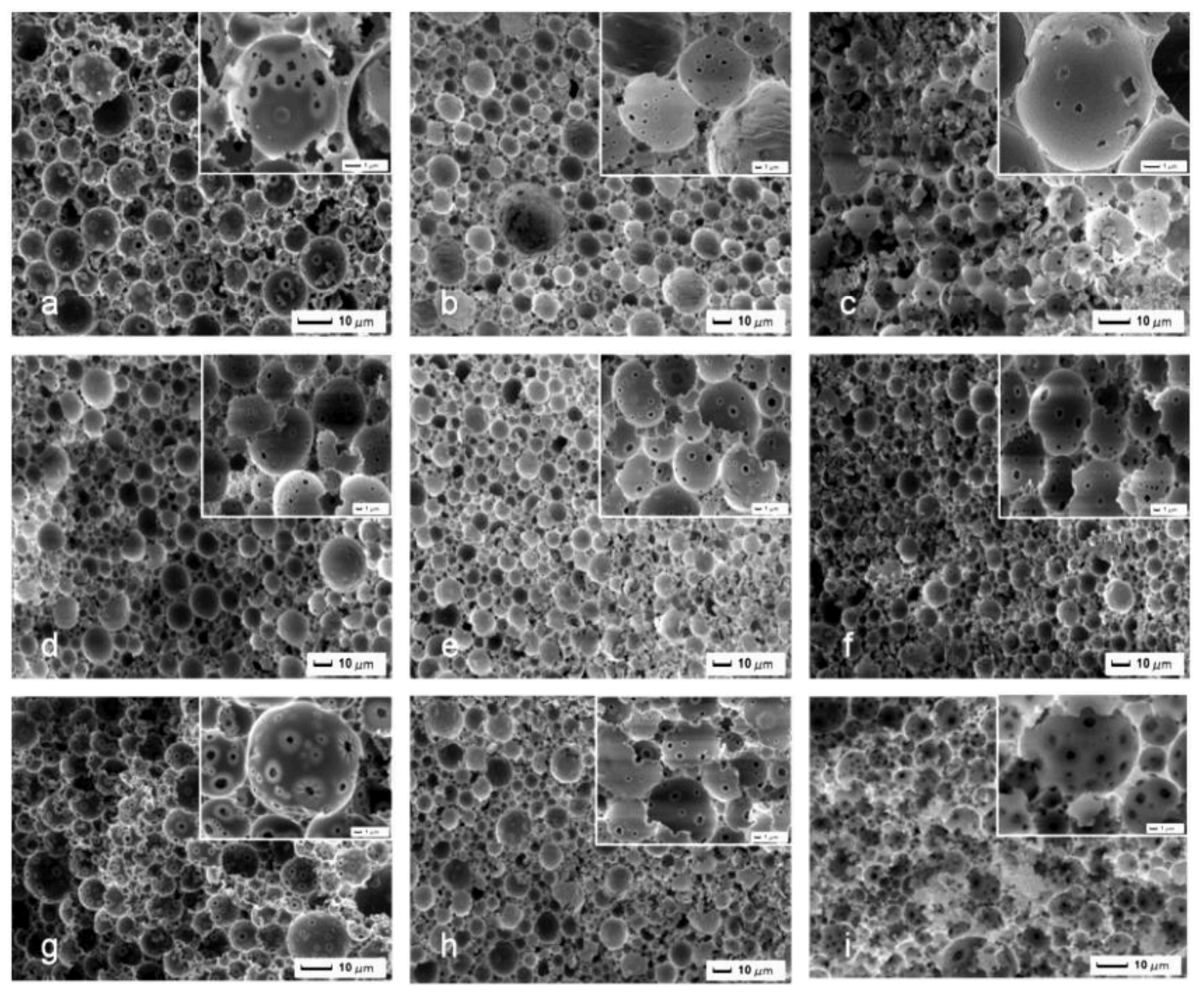
**SEM images of MMIPs 3, 5-10, 14, and 15, (a–c)** MMIPs 5, 7, and 14, **(d–f)** MMIPs 9, 6, and 10, **(g–i)** MMIPs 15, 8, and 3.

It was necessary to study the suitable amount of St/DVB as the continuous phase to improve the adsorption capacity. It was hypothesized that the concentration of St in the organic phase would increased the solubility of Hypermer 2,296 and therefore enhanced emulsion stability. The concentration of surfactant to the pre-made Pickering HIPEs was tested and studied its influence on pore and pore throats structure of the resulting macroporous polymer (Figure 4). MMIPs 12, 9, and 13 were therefore synthesized containing 13, 19, and 26 vol% Hypermer 2296 (with respect to the organic phase volume). In comparison to the open macroporous MMIPs, it was observed that higher number and significantly smaller diameter of pore throats per pore in MMIPs 12 than those of MMIPs 9 and 13. This might be explained that the decrease in pore size observed can be attributed to the decreased droplet size in the Pickering-HIPEs, which resulted in the increased viscosity in the presence of a higher concentration of Hypermer 2296. It was suggested that the surface sites per pore decreased when the pore throat increased, therefore both lower number and larger pore throat was not benefit for adsorption. The second series (~19 vol% surfactant) MMIPs 10, 9, and 11 had a relatively high adsorption capacity than those of the first series (~13 vol% surfactant) and the third series (~26 vol% surfactant), which resulted in the different polymerization degree for MMIPs due to different St concentration.

**Figure 5:**
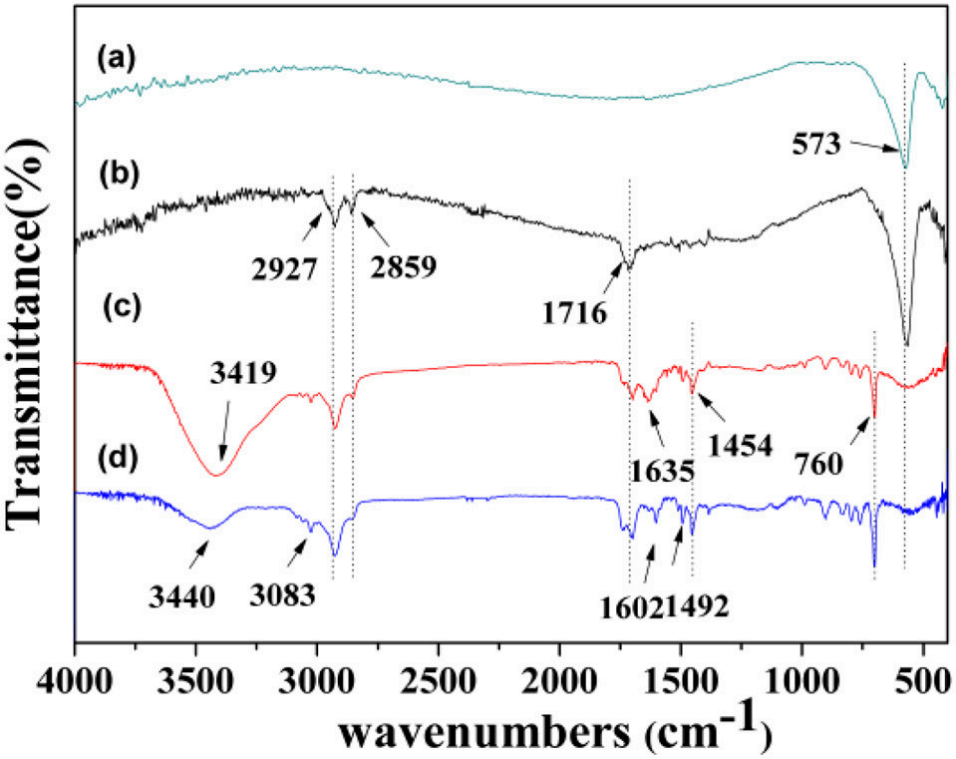
**FT-IR spectra of Fe_**3**_O_**4**_ (a)**, Fe_3_O_4_/OA **(b)**, MMIPs **(c)**, and MNIPs **(d)**.

The FT-IR spectra of Fe_3_O_4_ (a), Fe_3_O_4_/OA (b), MMIPs (c), and MNIPs (d) were obtained in Figure 5. The strong characteristic absorption band of Fe-O could be observed at around 573 cm^−1^, and the new peaks at 1,716 cm^−1^ (stretching vibrations of C = O for OA), 2,927 and 2,859 cm^−1^ (asymmetric stretching vibration of −CH_3_ and −CH_2_), indicated that Fe_3_O_4_ nanoparticles were successfully hydrophobic modified by OA. Compared with Fe_3_O_4_/OA, a broad absorption band at about 3,419 cm^−1^ of the MMIPs corresponded to the stretching vibration of O-H and C = C for MAA molecules (monomer); which was also obtained for MNIPs. The C = C stretching vibration peak at 1,635 cm^−1^ for St (monomer) and DVB (cross-linker) were also observed, other absorption bands, such as 3,083 cm^−1^ (stretching vibration of = C-H bonds of phenyl), 1,454 cm^−1^ (stretching vibration of residual vinylic C = C bonds), 760 cm^−1^ (bending vibration of = C-H bonds of phenyl), suggested that MMIPs was indeed coated onto the surfaces of the Fe_3_O_4_/OA for both MMIPs and MNIPS beads. The removal of LC, and the presence of the molecule-imprinted sites were confirmed by comparing curves (Figures 5c,d). The characteristic peak of C = C bond at 1,635 cm^−1^ was disappeared and the abroad peaks of OH at 3,440 cm^−1^ appeared after the elimination of the template, verifying the successful introduction of LC into the imprinted cavities.

**Figure 6:**
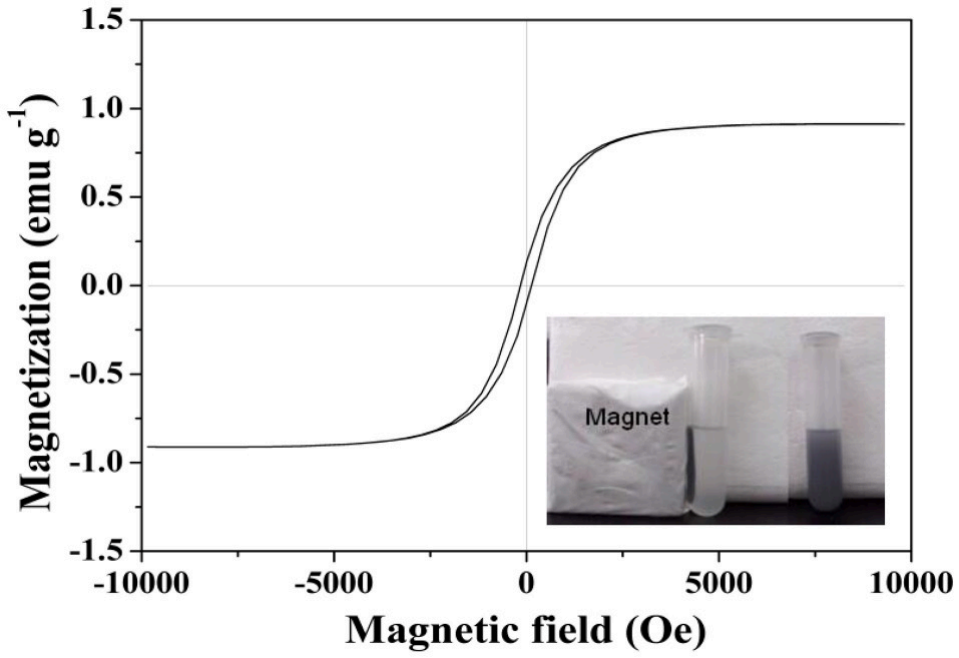
**Magnetizaiton curves obtained by VSM at room temperature of MMIPs, inset: the magnetic response of MMIPs to external magnetic field**.

VSM analysis was employed to study the magnetic property of MMIPs at room temperature. The magnetizaiton curves obtained of MMIPs was exhibited in Figure 6. The saturation magnetization (*M*_s_) value of MNIPs was 0.91 emu g^−1^. This result was indicated the MMIPs shell layer on the surface of Fe_3_O_4_/OA and the MMIPs can separate from aqueous solution quickly in practical application. Figure 6 (inset) also showed the separation and redispersion process of MMIPs. In the absence of an external magnetic field, a gray homogeneous dispersion formed. When an external magnetic field was applied, the gray particles were attracted to the wall of vial and the dispersion became clear and transparent. After the magnetic field was removed, the MMIPs could be re-dispersed rapidly by aggregating.

**Figure 7:**
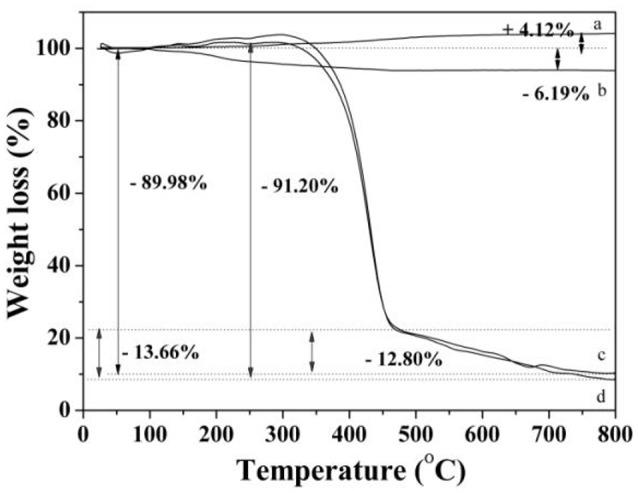
**The TGA curves of Fe_**3**_O_**4**_ (a)**, Fe_3_O_4_-OA **(b)**, MMIPs **(c)**, and MNIPs **(d)**.

TGA curves of the Fe_3_O_4_ (a), Fe_3_O_4_-OA (b), MMIPs (c), and MNIPs (d) were presented in Figure 7. According to the results, within the initial temperature range (< 350°C), the weight change was mainly due to the Fe_3_O_4_ nanoparticles which was oxidized to ferric oxide. With the temperature increased to 470°C, the significant weight losses of MMIPs (77.18%) and MNIPs (76.32%) could be observed which could be ascribed to the loss of carbohydrate in Fe_3_O_4_-OA and the polymer in MMIPs and MNIPs. In MMIPs and MNIPs curves, the difference of the weight loss may be due to the template molecules, which have different proportion between MMIPs and MNIPs. Above 450°C, the remaining mass for MMIPs and MNIPs might be attributed to the thermal resistance of the magnetic nanoparticles and residual carbon.

### Adsorption isotherm

**Figure 8:**
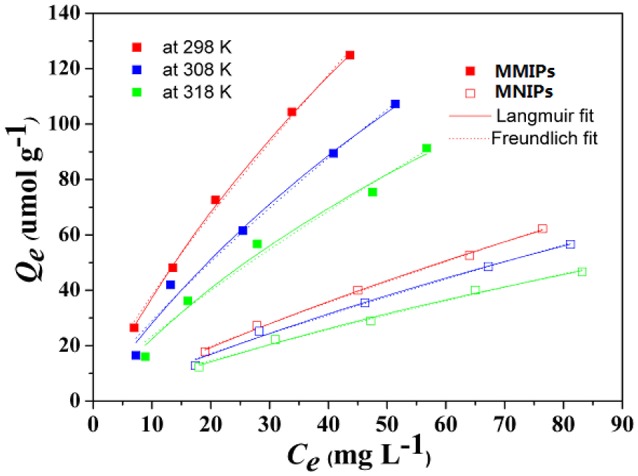
**The Langmuir and Freundlich isotherm models for LC using non-linear regression**.

Equilibrium adsorption isotherms were usually used to study the capacities of adsorbents from solution, it is vital to establish the equilibrium properties of the adsorption process by an appropriate coefficient (*R*^2^). Consequently, the equilibrium data of MMIPs and MNIPs for LC were fitted to the Langmuir (1918) and Freundlich (Levan and Vermeulen, 1981) isotherm models are presented in Figure 8. The non-linear forms of Langmuir isotherm model is expressed by the following equations (Liu et al., 2016):

(2) $$\begin{array}{r} Q_{e}=\frac{K_{L}Q_{m}C_{e}}{1+K_{L}C_{e}} \end{array}$$

Where *Q*_*e*_ (mg L^−1^) is the equilibrium adsorption capacity, *C*_*e*_ (mg L^−1^) is the equilibrium concentration of adsorbate at equilibrium, and *Q*_*m*_ (mg L^−1^) is the maximum adsorption capacity of the adsorbent. *K*_*L*_ (L mg^−1^) is the Langmuir adsorption constant.

For predicting the favorability of an adsorption system, the Langmuir equation can also be expressed in terms of a dimensionless separation factor R_*L*_ defined as follows (Langmuir, 1918):

(3) $$\begin{array}{r} R_{L}=\frac{1}{1+C_{m}K_{L}} \end{array}$$

where *C*_*m*_ is the maximal initial concentration of adsorbate. The *R*_*L*_ indicates the favorability and the capacity of adsorption system. When 0 < *R*_*L*_ < 1.0, it represents good adsorption.

The non-linear forms of Freundlich isotherm model is expressed by the following equations:

(4) $$\begin{array}{r} Q_{e}=K_{F}{C_{e}}^{\frac{1}{n}} \end{array}$$

*K*_*F*_ (mg L^−1^) is the Freundlich adsorption equilibrium constant, and 1/*n* is a measure of exchange intensity or surface heterogeneity, with a value of 1/*n* smaller than 1.0 describing favorable removal conditions (Levan and Vermeulen, 1981).

As can be seen from Figure 8, the maximum adsorption capacity for MMIPs and MNIPs were obtained using initial LC solution of 30–100 mg L^−1^, with the adsorption capacity of 404.4 and 272.96 μmol g^−1^, respectively. The adsorption capacity was increased with increasing the initial concentration because a high concentration difference provides a high driving force for the adsorption process. The adsorption capacity of the MMIPs was higher than that of MNIPS at every corresponding temperature. This result indicated that MMIPs have significant adsorption for LC than MNIPS, and the presence of the molecule-imprinted sites was confirmed and thus confirmed the successful imprinting processes via dummy template. The constants of adsorption of isotherm for LC were obtained and listed in Table 2.

**Table 2:** **Adsorption of isotherm constants and regression values for the adsorption of LC onto MMIPs and MNIPs**.

**Adsorption isotherm models**	**Constants**	**MMIPs**			**MNIPs**		
		**298 K**	**318 K**	**338 K**	**298 K**	**318 K**	**338 K**
Langmuir	*R*^2^	0.999	0.985	0.982	0.997	0.991	0.992
	*Q*_m,L_ (μmol g^−1^)	404.4	317.9	243.8	272.96	240.3	175.8
	*K*_L_ (L μmol^−1^)	0.01027	0.00977	0.01018	0.00382	0.004	0.004
	*R*_L_	0.145	0.158	0.13	0.145	0.158	0.13
Freundlich	*R*^2^	0.996	0.983	0.976	0.997	0.989	0.989
	*K*_F_ (L μmol^−1^)	6.041	4.675	3.935	1.541	1.331	1.23
	*R*^2^	0.806	0.796	0.777	0.8521	0.855	0.826

From the Table 2, it was concluded that the Langmuir model fitted better to the adsorption isotherm data, suggesting that LC adsorption on the surface of the MMIPs and MNIPs was a surface with homogeneous binding sites and monolayer coverage (Pan et al., 2015). It can also be seen that the values of *R*_*L*_ for MMIPs and MNIPs were 0.4933–0.5058 and 0.6944–0.7236 and *1/n* were 0.7766–0.8057 and 0.8265–0.8522 respectively, indicating that the MMIPs were favorable for LC adsorption than that of MNIPs.

### Adsorption kinetics

**Figure 9:**
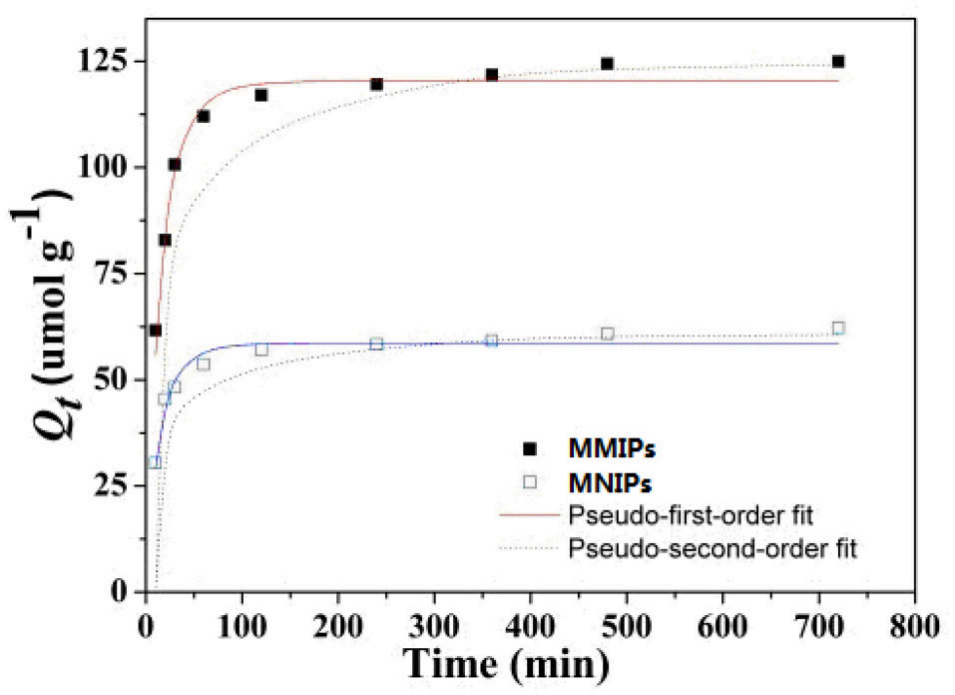
**Kinetic data for the adsorption of LC onto MMIPs and MNIPs**.

In order to understanding the adsorption kinetics and reveal the adsorption reaction mechanism, the batch contact time studies on the adsorption capacity of MMIPs and MNIPs were conducted at an initial LC concentration of 100 mg g^−1^, both the pseudo-first-order (Ho and McKay, 1999b) and pseudo-second-order (Ho and McKay, 1999a) models were fitted to experimental data were shown in Figure 9. The pseudo-first-order equation can be expressed as non-linear form by Equation (5):

(5) $$\begin{array}{r} Q_{t}=Q_{e}-Q_{e}e^{-k_{1}t} \end{array}$$

The pseudo-second-order equation can be expressed as non-linear form by Equation (6):

(6) $$Q_{t}=\frac{k_{2}{Q_{e}}^{2}t}{1+k_{2}Q_{e}t}$$

where *Q*_*t*_ (mg L^−1^) and *Q*_*e*_ (mg L^−1^) are the amount of LC adsorbed at time *t* and at equilibrium, respectively. *k*_1_ (L min^−1^) and *k*_2_ (g mg^−1^ min^−1^) are rate constants of the pseudo-first-order and pseudo-second-order models, respectively. Based on the pseudo-second-order kinetic rate constants, the initial adsorption rate (*h*, mg g^−1^ min^−1^) and half equilibrium time (*t*_1/2_, min) were also calculated according to the following equations (Pan et al., 2016a):

(7) $$\begin{array}{rcl} h & = & K_{2}{Q_{e}}^{2} \end{array}$$

(8) $$\begin{array}{rcl} t_{1/2} & = & \frac{1}{k_{2}Q_{e}} \end{array}$$

As can be seen from the Figure 9, the adsorption kinetic data of the samples were significantly increased with the beginning 60 min, and reached equilibrium capacity within 2.0 h. The kinetic constants for the adsorption of LC onto MMIPs and MNIPs by pseudo-first-order equation and preudo-second-order equation were summarized in Table 3. The pseudo-second-order kinetic model yielded a better fit than the pseudo-first-order model for the adsorption of LC onto MMIPs and MNIPs, indicating the chemical process could be the rate-limiting step in the adsorption process for LC (Yin et al., 2016; Pan et al., 2016b).

**Table 3:** **Pseudo-first-order and Pseudo-second-order adsorption kinetic constants and regression values of samples**.

**Adsorbent**	**Pseudo-first-order equation**				**Pseudo-second-order equation**				
	***Q*_e,exp_ (μmol g^−1^)**	***Q*_e,c_ (μmol g^−1^)**	***k*_1_ (min^−1^)**	***R*^2^**	***Q*_e,c_ (μmol g^−1^)**	***k*_2_ (min^−1^)**	***R*^2^**	***H* (μmol g^−1^ min^−1^)**	***t*_1/2_ (min)**
MMIPs	124.93	112.2	0.068	0.869	117.05	9.6 × 10^−4^	0.948	13.2	8.89
MNIPs	62.24	55.88	0.077	0.864	58.05	23 × 10^−4^	0.930	7.47	6.95

### Adsorption thermodynamics

In order to define whether the adsorption process is endothermic or exothermic and spontaneous, the thermodynamic parameters (Δ*H*°, Δ*S*°, and Δ*G*°) for LC adsorption on MMIPs and MNIPs were used to be calculated from the temperature dependent on adsorption isotherms. The standard enthalpy change (Δ*H*°) and the standard entropy (Δ*S*°) are then calculated from the linear plot of ln*K*° vs. 1/*T* for LC adsorption on the MMIPs and MNIPs by Equations (9, 10):

(9) $$\begin{array}{rcl} \ln K^{o} & = & \frac{\Delta S^{o}}{R}-\frac{\Delta H^{o}}{RT} \end{array}$$

(10) $$\begin{array}{rcl} K^{o} & = & \frac{Q_{e}}{C_{e}} \end{array}$$

where *R* is the universal gas constant (8.314 J mol^−1^ K^−1^), *T* is the temperature in Kelvin. *K*° is the adsorption equilibrium constant. The standard free energy change Δ*G*° Can be calculated by Equation (11):

(11) $$\begin{array}{r} \Delta {\text{G}}^{\text{o}}=\Delta H^{\text{o}}-T\Delta S^{\text{o}} \end{array}$$

The thermodynamic parameters of LC adsorption on MMIPs and MNIPs were listed in Table 4. The negative Δ*H*° value suggested that LC adsorption on the surface of the MMIPs and MNIPs was an exothermic procedure. The negative value of Δ*S*° suggested the decreased randomness at the solid-solution interface during the adsorption of LC on MMIPs and MNIPs. The negative Δ*G*° values obtained at 298 K exhibited that the adsorption process was spontaneous, whereas the Δ*G*° values increased with the temperature (at 298 K and 318 K) indicated that the adsorption of LC on the MMIPs and MNIPs was a non-spontaneous process when increased temperature. Thus, decreasing temperature was beneficial to the LC adsorption (Yu et al., 2008).

**Table 4:** **Thermodynamic parameters of LC adsorption on MMIPs and MNIPs**.

**Adsorbent**	***C*°(mg L^−1^)**	**Thermodynamic parameters**				***R*^2^**
		***T* (K)**	**Δ*G*° (kJ mol^−1^)**	**Δ*H*° (kJ mol^−1^)**	**Δ*S*° (J mol^−1^ K^−1^)**	
MMIPs	100	298	−0.61	−22.64	−82.25	0.998
		308	0.13			
		318	0.87			
MNIPs	100	298	2.46	−14.62	−57.33	0.987
		308	3.03			
		318	6.61			

### Adsorption selectivity

**Figure 10:**
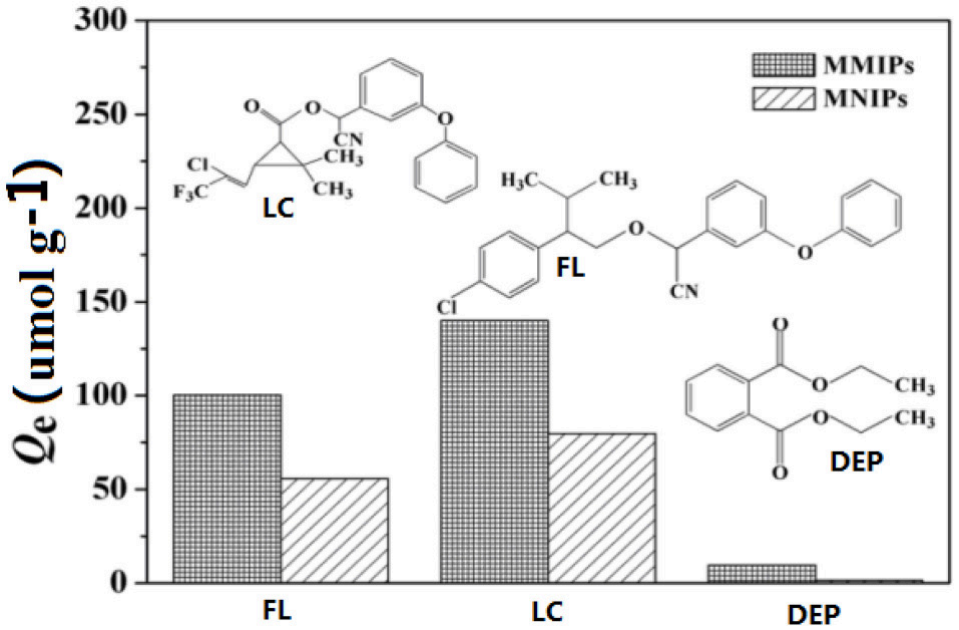
**Adsorption capacity of MMIPs and MNIPs for LC, FL, and DEP, insert: the structure of the test compounds**.

To investigate the adsorption selectivity of the MMIPs, the adsorption experiments of MMIPs and MNIPs for LC, structural analog FL and non-analog DEP were carried individual at the same concentration of 100 mg L^−1^ in aqueous solutions at, and the chemical structures were listed in Figure 10. As a comparison, the adsorption capacity of MMIPs and MNIPs suggested that MMIPs was specific for LC and FL and non-specific to DEP. This phenomenon suggested that the binding sites have special recognition ability to target molecules due to hydrogen bonds could form between the adsorbates and adsorbents. FL not only has the similar structure groups but the lower molecular weight, although the adsorption capacity was higher than that of LC, the difference (60.96) between MMIPs and MNIPs of LC was higher than that of FL (54.68), this could indicated that the MMIPs have specific recognition ability. However, DEP despite has small molecular, there were no hydrogen bond and it was too weak for molecules to get into the cavities which resulted very low adsorption capacity, it should implied that the hydrogen bond played an important role for adsorption.

### Regeneration analysis

**Figure 11:**
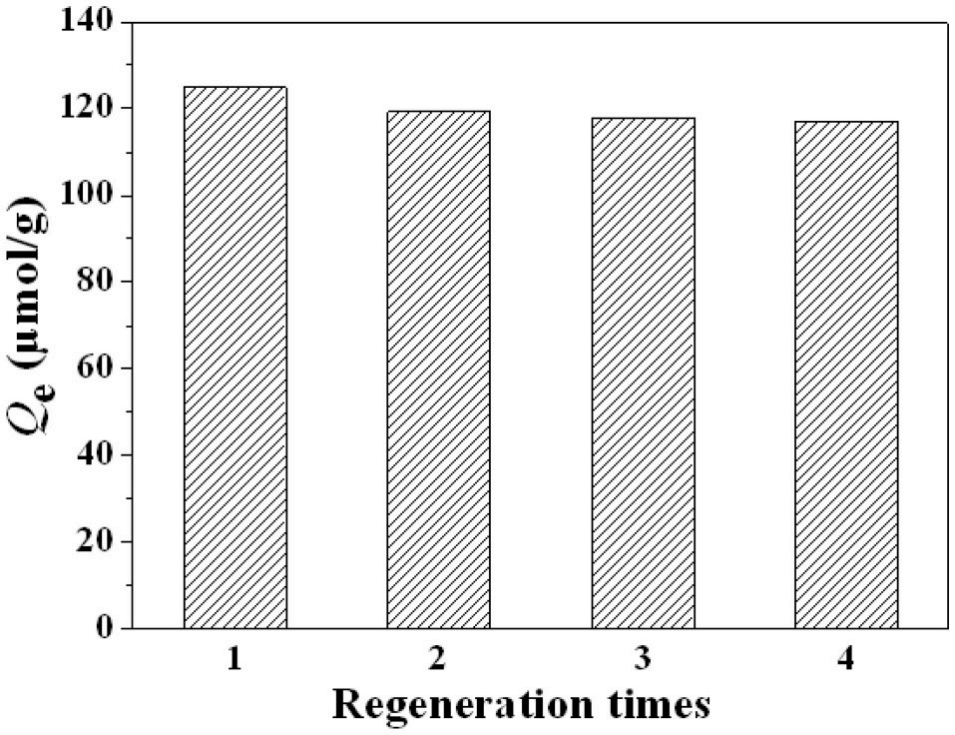
**Regeneration of the MMIPs for four cycles**.

The regeneration of adsorbent, a vital factor on its application, was investigated for four sequential cycles of adsorption-desorption. The regenerated results were shown in Figure 11, the adsorption capacity of MMIPs for LC still remained a high recovery over 85% after the four cycles. It is concluded that recognizing sites were stable and the adsorbent could be reused after regeneration in applications.

## Conclusions

A novel MMIPs used for recognizing and capture LC was successfully synthesized via W/O particle-stabilized HIPEs, and the it was investigated to the selective adsorption LC from aqueous solution. The adsorption equilibrium data yielded a better fit to Langmuir model and kinetic data followed the pseudo-second-order model. In addition, the resulting MMIPs proved significant selectivity and good repeatability for adsorption of LC molecules in aqueous solution.

## Author contributions

YW and RG contributed to the experimental studies and manuscript preparation. JL contributed to the data analysis. YM and JP contributed to the study design, manuscript revision, and final version approval.

### Conflict of interest statement

The authors declare that the research was conducted in the absence of any commercial or financial relationships that could be construed as a potential conflict of interest.
